# Declines in Malaria Burden and All-Cause Child Mortality following Increases in Control Interventions in Senegal, 2005–2010

**DOI:** 10.4269/ajtmh.16-0953

**Published:** 2017-09-27

**Authors:** Julie Thwing, Erin Eckert, Demba Anta Dione, Roger Tine, Adama Faye, Yazoume Yé, Medoune Ndiop, Moustapha Cisse, Jacques Andre Ndione, Mame Birame Diouf, Mady Ba

**Affiliations:** 1 President's Malaria Initiative, Malaria Branch, Centers for Disease Control and Prevention, Atlanta, Georgia;; 2 President's Malaria Initiative, U.S. Agency for International Development, Washington, District of Columbia;; 3 Health and Development Solutions-Africa, Dakar, Senegal;; 4 Université Cheikh Anta Diop, Dakar, Senegal;; 5 MEASURE Evaluation/ICF International, Rockville, Maryland;; 6 National Malaria Control Program, Dakar, Senegal;; 7 Centre de Suivi Ecologique, Dakar, Senegal;; 8 U.S. Agency for International Development, Dakar, Senegal

## Abstract

Malaria is endemic in Senegal. The national malaria control strategy focuses on achieving universal coverage for major interventions, with a goal of reaching preelimination status by 2018. Senegal began distribution of insecticide-treated nets (ITNs) and introduced artemisinin-based combination therapy in 2006, then introduced rapid diagnostic tests in 2007. We evaluated the impact of these efforts using a plausibility design based on malaria’s contribution to all-cause under-five mortality (ACCM) and considering other contextual factors which may influence ACCM. Between 2005 and 2010, household ownership of ITNs increased from 20% to 63%, and the proportion of people sleeping under an ITN the night prior to the survey increased from 6% to 29%. Malaria parasite prevalence declined from 6% to 3% from 2008 to 2010 among children under five. Some nonmalaria indicators of child health improved, for example, increase of complete vaccination coverage from 58% to 64%; however, nutritional indicators deteriorated, with an increase in stunting from 16% to 26%. Although economic indicators improved, environmental conditions favored an increase in malaria transmission. ACCM decreased 40% between 2005 and 2010, from 121 (95% confidence interval [CI] 113–129) to 72 (95% CI 66–77) per 1,000, and declines were greater among age groups, epidemiologic zones, and wealth quintiles most at risk for malaria. After considering coverage of malaria interventions, trends in malaria morbidity, effects of contextual factors, and trends in ACCM, it is plausible that malaria control interventions contributed to a reduction in malaria mortality and to the impressive gains in child survival in Senegal.

## INTRODUCTION

**Figure 1.**
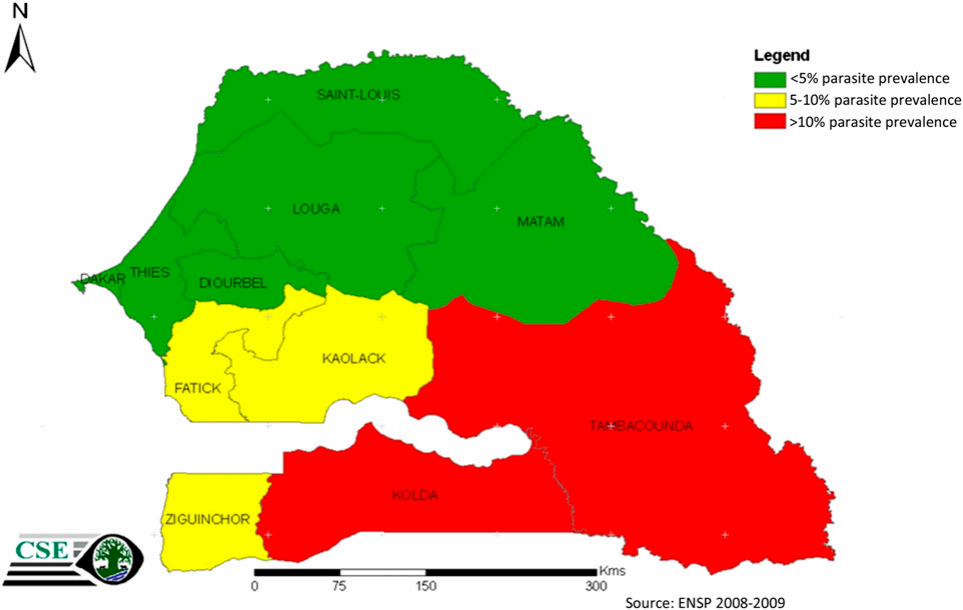
Epidemiologic stratification by parasite prevalence, Senegal 2009.

Malaria is endemic throughout Senegal, where 100% of the population is at risk of infection, and has historically been a leading cause of morbidity and mortality across the country. Senegal is split into four malaria epidemiological zones based on parasite prevalence. Malaria epidemiological zones are characterized by very low transmission in the north, low transmission in the center, and moderate transmission in the south (Figure 1). In the capital, Dakar, malaria transmission is very heterogeneous, representing a separate epidemiological zone. According to routine data collected by the National Malaria Control Program (NMCP) in 2010, annual incidence of confirmed malaria cases reported by health facilities ranged from less than 5/1,000 persons in the Sahelian north to greater than 100/1,000 persons in the more tropical southeast. *Plasmodium falciparum* is the major malaria parasite species, accounting for 99% of all infections, with *Plasmodium malariae* and *Plasmodium ovale* making up the remaining 1%. The main vector species are *Anopheles gambiae* s.s., *Anopheles arabiensis*, *Anopheles funestus*, and *Anopheles melas*, depending on rainfall and the presence of permanent sources of water.

Although the Ministry of Health has supported malaria control efforts for many years, the advent of the Roll Back Malaria (RBM) Partnership in 1998 galvanized the NMCP into developing a series of coordinated plans and intervention programs. The country’s current strategy, with a goal of reaching preelimination status by 2018, is focused on achieving universal coverage of effective malaria interventions: insecticide treated nets (ITNs) and/or indoor residual spraying (IRS), intermittent preventive (IPTp), malaria diagnostic testing, predominantly through rapid diagnostic tests (RDTs), and appropriate treatment of all malaria cases with recommended therapy, artemisinin-based combination therapies (ACTs). Seasonal malaria chemoprevention and reactive case detection have been recently introduced in specific geographic areas.

**Figure 2.**
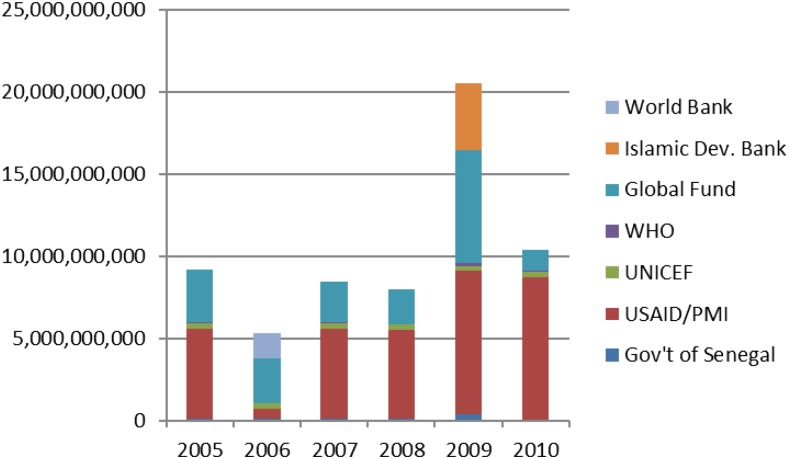
Financial contributions to malaria control in CFA, Senegal 2005–2010. (Source: Senegal National Strategic Framework for Malaria, 2014–2018).

Senegal progressively introduced these interventions over several years. The NMCP began to accelerate malaria control implementation in 2006 by increasing the availability of highly subsidized ITNs and the introduction of ACT. Introduction of RDTs followed in 2007 and they, like ACTs, were rolled out first at the health facility level and then at the community level (Table 1). The introduction of RDTs led to changes in malaria case definition, from a clinical diagnosis based on symptoms alone, to one based on both clinical assessment and parasitological confirmation. IRS also started in 2007 in three districts and increased to six districts in 2010, covering approximately 8% of the national population. In 2008, the NMCP introduced home-based management of malaria (PECADOM) with RDTs and ACTs in remote villages, and scaled up to 841 villages by 2010. Also in 2008, a subnational distribution of long-lasting insecticidal nets (LLINs) targeting children under-five was conducted, followed by a nationwide distribution campaign targeting the same age group in 2009. In 2010, the NMCP adopted a strategy of universal coverage of LLINs, and the four highest transmission regions (in southern Senegal) were the first to be targeted for universal coverage during mass LLIN distribution in 2010. During the 6 year period from 2005 to 2010, a total combined budget of $103 million was committed to malaria control from government, multilateral, and bilateral sources (Figure 2).

**Table 1:** ITNs, RDTs, ACTs distributed annually in Senegal, 2005–2010

	2005	2006	2007	2008	2009	2010
ITNs	599,689	849,888	1,076,141	1,586,522	2,532,018	1,258,663
RDTs	0	148,000*	374,225	625,775	1,041,925	1,252,900
ACTs	0	2,281,609	990,141	743,611	704,367	961,884

Source: NMCP Senegal. ACT = artemisinin-based combination therapy dose; ITN = insecticide-treated net; RDT = rapid diagnostic test kit.

* This figure represents RDTs being piloted in Senegal prior to the official policy regarding their use being implemented in 2007.

The introduction of RDTs, and the requirement of parasitological testing to confirm cases, resulted in a pronounced decrease in the number of cases of malaria detected and reported from health facilities. The number of ACT treatments prescribed also decreased as confirmatory testing reduced overprescription of ACTs. At the health facility level, a suspected malaria case is defined as a patient with fever or history of fever without symptoms indicative of another febrile illness. The proportion of suspected malaria cases tested at health facilities reached 80% within a year of RDT introduction. From 2008 on, the number of cases treated with ACTs at health facilities closely tracked the number of cases confirmed by RDT.^1^

The period 2005–2010 also saw a number of efforts aimed at improving maternal and child health beyond those focused on malaria control. The Ministry of Health and its partners supported a number of interventions including the treatment of diarrhea with oral rehydration salts (ORS) and zinc, expanded immunization coverage, and improved access to and use of family planning. In addition to these ongoing initiatives, Senegal also conducted a number of mass campaigns such as polio immunization days, administration of antihelminthics, and vitamin A supplementation.

In conjunction with the RBM Partnership, the President’s Malaria Initiative, Senegal’s NMCP, and other partners conducted an evaluation of the impact of national malaria control efforts for the period 2005 to 2010, given the time frame of the rapid scale-up of malaria control interventions and availability of high quality survey data. The objective of the evaluation was to assess whether a decline in malaria-related morbidity and all-cause under-five mortality (ACCM) could be plausibly associated with malaria control activities.^2^

## METHODS

### Evaluation design.

As recommended by the RBM Monitoring and Evaluation Reference Group,^3^ the evaluation is based on a before-and-after assessment, which uses a plausibility evaluation design that measures changes in malaria intervention coverage, malaria-related morbidity, and all-cause mortality while considering other contextual determinants of child survival to plausibly attribute the impact of malaria control interventions on ACCM. The rationale of this evaluation design, impact measures and indicators are discussed elsewhere in this supplement.^4^

### Evaluation timeline.

The timeframe selected for this evaluation was the period 2005–2010, representing the period of rapid increase of malaria control interventions and corresponding to the Ministry of Health’s Strategic Plan for Malaria Control 2006–2010.^5^ Four national household surveys were conducted at several points during this time period (2005, 2006, 2008, 2010) to monitor trends in intervention coverage and evaluate the impact on morbidity and mortality. Mortality is calculated over the previous 5 years, approximating mortality at the midpoint of the period. Although a continuous Demographic and Health Survey (DHS) was started in 2012, no data were available at the time of this evaluation.

### Data sources and definitions.

In Senegal, the major effort to scale-up malaria control interventions began in 2006. The preintervention period is defined as the period 2000–2005 for which data on mortality among children under five are available for the five preceding years (2000–2005)^6^ from the DHS conducted in 2005. The intervention period is defined as the period from 2006 to 2010, when malaria control interventions were implemented nationwide. Data on mortality among children under-five for this period are obtained from the DHS conducted during 2010–2011.^7^ The main sources of data for this assessment, therefore, are these two DHS, as well as two Senegal Malaria Indicator Surveys (MIS), conducted in 2006^8^ and 2008.^9^ The basic morbidity measures for malaria burden are severe anemia and parasite prevalence among children 6–59 months of age. Changes in malaria-related anemia over time are tracked by the prevalence of severe anemia (hemoglobin concentration < 8 g/dL).

Both DHS and MIS also provided information regarding intervention coverage and contextual factors (factors which may influence transmission of malaria or ACCM). These surveys also provided the information on the wealth quintiles, an approximation of the socioeconomic status of the households.^10^ ITN coverage measures from surveys included household ITN ownership, population access to an ITN, and population sleeping under an ITN, as well as a combined vector control intervention measure of household ITN ownership or IRS. IRS was not considered independently as it was implemented in a limited number of districts. Malaria diagnostic testing was assessed through the proportion of febrile children who underwent a finger or heel stick. Malaria treatment coverage was assessed by the proportion of febrile children who received any antimalarial and the proportion who received an ACT. Contextual factors included interventions for other conditions (ORS, immunizations, breastfeeding, and vitamin A supplementation), prevalence of other diseases (pneumonia and diarrhea), and nutritional status indicators (stunting, underweight, and wasting).

In addition, the evaluation used data from the NMCP’s routine malaria information system to examine temporal trends in malaria morbidity and mortality. This system compiles data on the number of total patients, suspected cases, tested cases, confirmed cases, hospitalized cases of confirmed malaria, and deaths due to confirmed malaria reported to the NMCP on a quarterly basis by public health facilities, including hospitals. These data are further validated through a system of quality checks during supervision and through quarterly peer review. Incidence of malaria per thousand (unadjusted for consultation, testing, or reporting rates) is defined as the total number of confirmed cases of malaria reported by health facilities in each district divided by the estimated population of each district, multiplied by 1,000. Proportional malaria mortality is defined as the number of deaths in hospitalized patients with confirmed malaria divided by all in-hospital deaths. Malaria case fatality rate is defined as number of deaths among inpatients with confirmed malaria divided by the number of confirmed malaria hospitalizations.

The evaluation also considered contextual factors using data from a variety of supplemental sources. Some of the additional data sets included:

- Data regarding other maternal and child health intervention coverage derived from the large national surveys (DHS, MIS);
- Data regarding timing, targeting, and financing of interventions from the NMCP and partners.
- Data regarding environmental conditions (Weighted Anomaly Standardization Precipitation [WASP] and Normalized Difference Vegetation Index [NVDI] from the *Center de Suivi Ecologique.*

## RESULTS

### Trends in Intervention coverage from 2005 to 2010.

#### Vector control.

**Figure 3.**
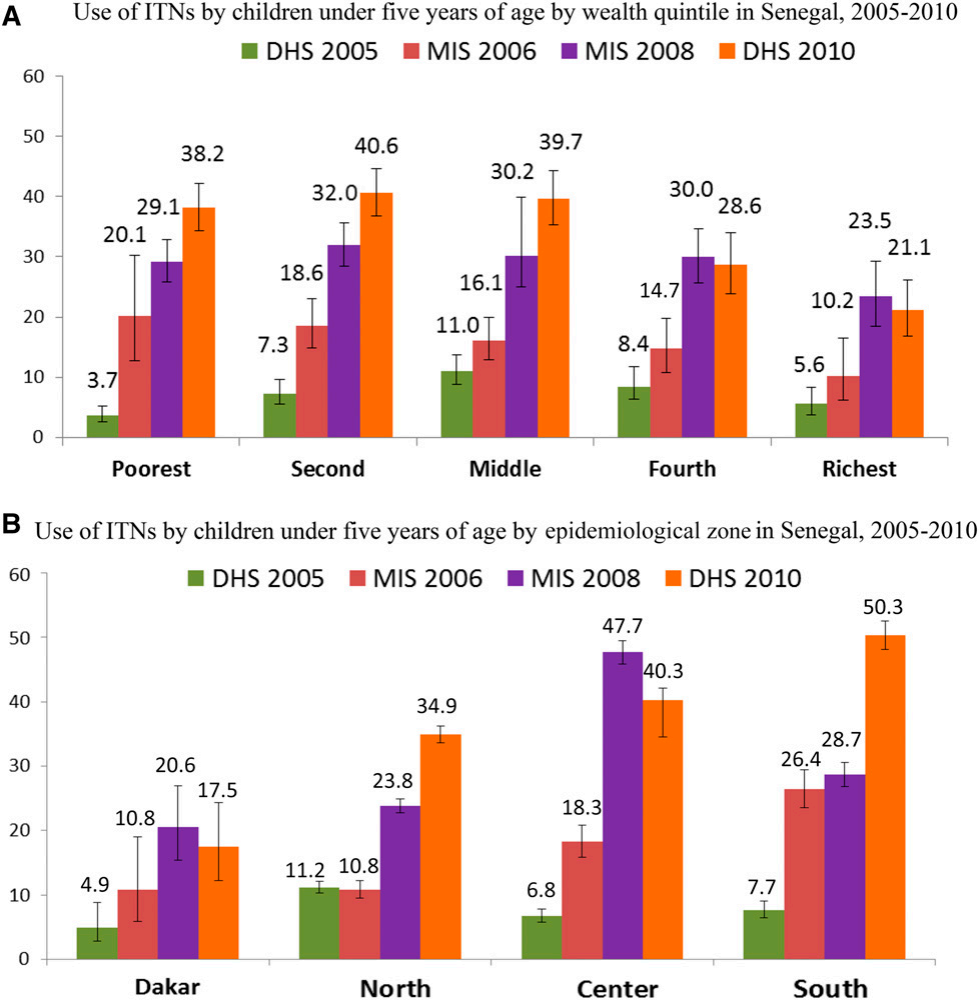
(**A**) Use of insecticide-treated nets (ITNs) by children under-five by wealth quintile in Senegal, 2005–2010. (**B**) Use of ITNs by children under-five by epidemiological zone in Senegal, 2005–2010.

The proportion of households protected by either an ITN or IRS increased from 37% in 2006 (the first year IRS coverage was measured) to 66% in 2010. Ownership of at least one ITN by households increased from 20% in 2005, to 36% in 2006, 60% in 2008, and 63% in 2010, a significant increase from 2005 to 2010 (*P* < 0.001). Results stratified by socioeconomic quintile and epidemiologic zone showed that increases in ITN ownership were greatest among the poorest quintiles and in the highest transmission zones (Figure 3). The proportion of the population with access to an ITN in the household increased from 10% in 2005 to 38% in 2010. The increase in ITN household ownership and population access was accompanied by an increase in the proportion of the population sleeping under an ITN. The proportion of the general population that slept under an ITN increased from 6% in 2005 to 29% in 2010 (Table 2). The proportion of children under-five sleeping under an ITN similarly increased substantially, from 7% in 2005, to 20% in 2006, to 32% in 2008, and to 35% in 2010 (*P* < 0.001). IRS was never scaled beyond six pilot districts, which received the same ITN distributions as non-IRS districts.

**Table 2:** Household ownership of, population sleeping under, and population access to ITNs by the general population (all ages) in Senegal, 2005–2010

Survey	% Of HH with at least one ITN	% Of population sleeping under an ITN the previous night	% Of population with access to an ITN in HH*	Ratio use: access
DHS 2005	20.3	5.8	9.8	0.61
MIS 2006	36.6	12.2	17.5	0.69
MIS 2008	60.4	22.9	34.9	0.66
DHS 2010	62.9	28.9	38.1	0.76

ITN = insecticide-treated net; IMS = Malaria Indicator Surveys; HH = household.

* Proportion of the population who could have slept under an ITN the night before the survey, in households with nets for every 2 people. (http://www.rollbackmalaria.org/files/files/resources/tool_HouseholdSurveyIndicatorsForMalariaControl.pdf).

#### Malaria during pregnancy.

Overall, coverage of interventions for prevention of malaria during pregnancy improved from 2005 to 2010. The proportion of pregnant women who received two doses of sulfadoxine–pyrimethamine (SP) as IPTp treatment during pregnancy (IPTp) increased from 12% in 2005 to 49% in 2006, and 52% in 2008. Coverage decreased to 39% in 2010 due to a nationwide stock-out of SP in 2009, but overall increased (*P* < 0.001) during the timeframe under consideration. Use of ITNs by pregnant women also increased between 2005 and 2010, from 9% in 2005 to 17% in 2006, 30% in 2008, and 36% in 2010 (*P* < 0.001).

#### Case management.

Among mothers who reported their children had fever in the last 2 weeks, the proportion that reported their child had a finger or heel stick, a proxy for received a malaria diagnostic test, increased from 5% in 2008 to 10% in 2010 (*P* = 0.001). Increases were significant across epidemiologic zones, socioeconomic status, and urban/rural residence, although actual percentages remained fairly low. The greatest increases were among the wealthiest quintile (4–14%), in Dakar (4–12%), and among urban dwellers (4–10%), but the testing rate in the high transmission southern zone was the same as Dakar in 2010 (12%) (Table 3).

**Table 3:** Proportion of children under five years with fever during the previous 2 weeks who received a finger or heel stick blood test at any point

Characteristics	MIS 2008		DHS 2010		Variation (95% CI)	*P* value
	% (95% CI)	*N*	% (95% CI)	*N*		
Total	4.9 (4.0–5.9)	4,123	9.7 (8.0–11.8)	2,463	4.8 (3.4–6.1)	0.001
Epidemiologic zone						
Dakar	3.7 (2.3–6.0)	1,116	12.0 (7.8–17.9)	800	8.3 (5.8–10.8)	0.001
North	4.1 (3.3–5.1)	1,613	7.4 (5.7–9.0)	965	3.3 (1.4–5.2)	0.001
Center	7.9 (5.7–10.1)	577	9.1 (6.1–12.1)	353	1.2 (–2.5;4.9)	0.26
South	5.7 (4.1–7.2)	817	11.6 (8.2–14.9)	344	5.9 (2.2–9.6)	0.001
Socio–economic quintile						
Poorest	5.0 (3.7–6.7)	901	8.0 (5.7–11.0)	490	3.0 (0.2–5.7)	0.01
Fourth	5.3 (3.8–7.4)	808	7.7 (5.3–10.9)	390	2.4 (–0.6–5.4)	0.05
Middle	5.3 (3.6–7.7)	796	7.8 (5.5–11.0)	430	2.5 (–0.4–5.4)	0.04
Second	4.4 (2.8–6.9)	825	9.9 (7.1–13.8)	626	5.5 (2.7–8.2)	0.001
Least poor	4.4 (2.5–7.4)	793	14.2 (8.8–22.2)	528	9.8 (6.5–13.1)	0.001

Source: MIS 2008, DHS 2010. N = number of children under five.

Among children with fever in the previous 2 weeks, receipt of any antimalarial declined from 27% in 2005 to 20% in 2006, 9% in 2008, and 8% in 2010. Similar to the findings regarding diagnostic testing, residents of Dakar, and those in the highest socioeconomic quintile marked the highest use of any antimalarial. The proportion of children with fever who received the first-line antimalarial (an ACT after 2006) decreased from 8% in 2005 to 3% in 2010, with the same trends by epidemiologic zone and wealth quintile, consistent with increased use of confirmatory testing by RDT, in the context of relatively low malaria incidence.

### Change in malaria related morbidity.

#### Data from household surveys.

As measured in the household surveys, prevalence of severe anemia in children under five decreased from 20% in 2005 to 14% in 2010 (*P* < 0.001). Significant decreases were noted across all wealth quintiles and all epidemiologic zones except the south. Malaria parasite prevalence among children under five also decreased, from 6% in 2008 (the first year it was measured) to 3% in 2010. It decreased significantly among the poorest two wealth quintiles. A reduction in malaria parasite prevalence was noted in all zones except Dakar, with the greatest decrease in the south (high transmission), followed by the center (moderate transmission). Although parasite prevalence did not decline significantly among children 6–11 months, it decreased significantly among children 12–23 months and 24–59 months (Table 4).

**Table 4:** Malaria parasite prevalence in children 6–59 months confirmed by microscopy in Senegal, 2008 and 2010

Characteristics	MIS 2008		DHS 2010		Variation (95% CI)	*P* value*
	% (95% CI)	*N*	% (IC 95%)	*N*		
Total	5.7 (4.4–7.3)	3,847	2.9 (2.1–3.8)	3,762	−2.8 (−3.7; −1.8)	0.001
Age (months)						
6–11	2.4 (1.3–4.6)	340	1.9 (0.9–3.8)	394	−0.5 (−2.6; 1.6)	0.32
12–23	3.5 (2.3–5.4)	863	1.4 (0.8–2.6)	813	−2.1 (−3.5; −0.6)	0.002
24–59	6.8 (5.8–7.7)	2,644	3.4 (2.6–4.1)	2,553	−3.4 (–4.5; −2.2)	0.001
Epidemiologic zone						
Dakar	0.8 (0.2–3.2)	723	1.5 (0.4–6.2)	761	0.7 (−0.3; 1.7)	0.10
North	2.4 (1.6–3.1)	1,769	1.1 (0.6–1.6)	1,528	−1.3 (−2.1; −0.4)	0.002
Center	6.9 (5.1–8.6)	803	3.7 (2.4–4.9)	873	−3.2 (−5.3; −10.4)	0.001
South	20.6 (17.2–23.9)	552	7.7 (0.4–6.2)	600	−12.9 (−16.9; −8.9)	0.001
Wealth quintile						
Poorest	15.8 (12.1–20.4)	855	6.2 (4.2–8.9)	849	−9.6 (6.6; 12.5)	0.001
Fourth	7.3 (4.7–11.4)	843	2.1 (1.2–3.5)	817	−5.0 (−8.7; −1.7)	0.001
Middle	1.4 (0.7–2.9)	825	1.6 (0.8–3.3)	764	0.2 (−1.5; 5.5)	0.86
Second	0.7 (0.2–2.1)	709	1.6 (0.7–3.7)	734	0.9 (0.5; 1.2)	0.001
Richest	0.7 (0.2–3.0)	615	2.3 (0.7–7.3)	597	1.6 (1.3; 4.5)	0.001

Source: DHS 2010, MIS 2008. N = number of children under-five (denominator).

* Pearson χ^2^ one-sided test.

#### Data from routine information systems.

**Figure 4.**
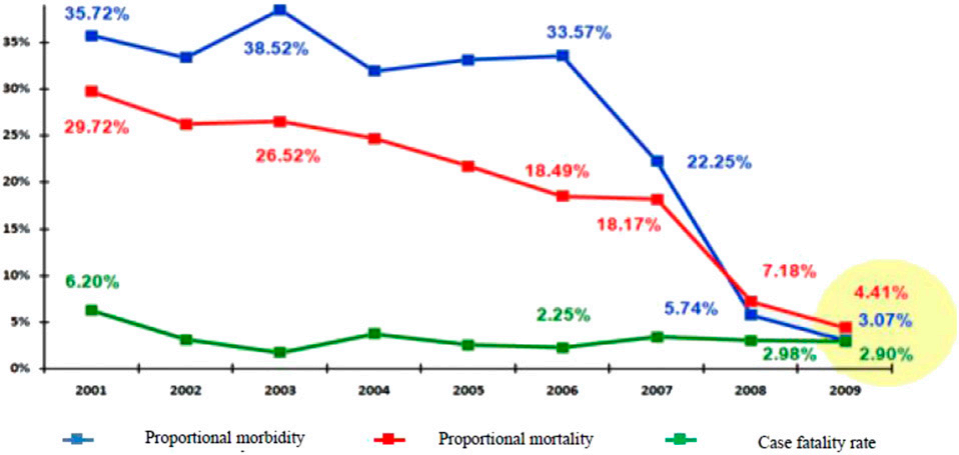
Proportional morbidity, proportional mortality, and case fatality rate at health facilities in Senegal, 2001–2009. Proportional morbidity: proportion of all-cause consultations associated with parasitologically confirmed malaria. Proportional mortality: proportion of all hospitalized deaths associated with parasitologically confirmed malaria. Case fatality rate: proportion of hospitalized cases of parasitologically confirmed malaria for whom death is the outcome.

Although the nationwide annual incidence of confirmed malaria illness episodes per thousand persons remained unchanged from 2008 (the first year after RDT introduction) to 2010 at 20/1,000 per year, there were important regional differences. Although Dakar, northern, and central zones reported modest increases of 1–6 per thousand from 2008 to 2010 (a year of record rainfall) the incidence of malaria illness in the high transmission southern zone decreased from 63/1,000 to 37/1,000. All epidemiologic zones except Dakar noted a decrease from 2008 to 2009, with all but the southern zone increasing from 2009 to 2010 (Table 5), consistent with the timing and locations of net distributions nationwide to children under five years in 2009 and for all sleeping spaces (a universal coverage strategy) in the southern zone in 2010. The proportion of all consultations due to malaria decreased from 34% in 2005 (pre-RDT introduction) to 3% in 2009 (Figure 4).

**Table 5:** Laboratory-confirmed* malaria illness episodes incidence per thousand among children under five by epidemiologic zone, in Senegal, 2008–2010

Epidemiologic zone	2008	2009	2010
Total	20.7	13.4	19.8
Dakar	11.9	11.8	17.5
North	10.7	6.6	11.8
Center	21.2	9.1	26.2
South	62.9	40.2	37.0

Source: NMCP Senegal.

* Cases confirmed by RDT in outpatients and microscopy in inpatients, the majority being confirmed by RDT.

### Change in all-cause child mortality.

#### Data from household surveys.

ACCM decreased 40% between 2005 and 2010, from 121 per 1000 (95% confidence interval [CI] 113.3–129.1) to 72 per 1000 (95% CI 65.8–77.3) live births. ACCM decreased among all wealth quintiles, with significant decreases found in the poorest three quintiles, with decreases of 41% (162–96 per 1,000 live births), 48% (144–74), and 41% (117–69) in the poorest, fourth, and middle wealth quintiles, respectively (*P* < 0.001 for all). Significant decreases were observed in all epidemiologic zones, but the greatest decreases were in the zones with the highest malaria transmission: the center (45%, from 123 to 68 deaths per 1,000 live births) and the south (42%, from 165 to 96 deaths per 1,000 live births). Mortality decreased significantly among all age bands except neonatal (0–28 days), with the greatest decreases among children 24–59 months (59%) and 12–23 months (57%) (Table 6).

**Table 6:** All-cause mortality among children under five per 1,000 live births in Senegal 2005–2010

Characteristics	DHS 2005		DHS 2010		Variation (%)	*P* value
	Deaths per 1,000 live births (95% CI)	N	Deaths per 1,000 live births (95% CI)	N		
Total	121.2 (113.32–129.14)	19,052	71.6 (65.8–77.34)	19,992	40.1	< 0.001
Age band						
6–11 months	13.7 (11.9–15.3)	17,983	7.4 (6.2–8.7)	19,116	46.0	0.001
12–23 months	18.6 (16.7–20.8)	17,770	8.1 (6.4–9.5)	18,977	56.5	0.001
24–59 months	30.0 (27.5–32.7)	17,844	12.2 (10.7–14.7)	18,823	59.3	0.001
Neonatal (0–28 days)	34.6 (30.4–39.5)	19,052	29.2 (25.48–33.4)	19,992	15.6	0.220
Post–neonatal (1–12 months)	26.4 (23.8–30.8)	18,295	17.5 (15.1–21.0)	19,357	33.7	0.001
Infant (0–12 months)	61.0 (55.4–66.6)	19,052	46.7 (41.9–51.5)	19,992	23.4	0.001
Epidemiologic zone						
Dakar	79 (69.9–88.1)	3,374	55.0 (47.7–62.3)	3,770	30.4	0.001
North	100 (93.4–106.5)	8,021	68.0 (62.6–73.4)	8,442	32.0	0.001
Center	123 (113.2–132.7)	4,338	68.0 (60.6–75.3)	4,500	44.7	0.001
South	165 (152.4–177.6)	3,320	96.0 (85.9–52.8)	3,280	41.8	0.001
Socioeconomic quintile						
Poorest	162.3 (145.5–178.7)	4,501	96.2 (84.4–100.7)	4,829	40.7	0.001
Fourth	144.0 (129.4–158.3)	4,328	74.2 (63.7–84.7)	4,448	48.4	0.001
Middle	117.2 (99.5–134.5)	3,963	68.7 (56.0–81.3)	3,868	41.3	0.001
Second	89.9 (69.7–109.6)	3,473	64.4 (46.0–82.4)	3,802	28.3	0.084
Richest	68.8 (48.0–91.0)	2,788	43.2 (24.8–61.2)	3,006	37.2	0.062

Source: DHS 2005, 2010. *N* = number of children.

#### Data from the routine information systems.

From 2001 to 2009, malaria proportional mortality decreased from 30% to 4%, likely reflective of both the improved coverage of interventions as well as the roll out of diagnostic testing. During the same period, the case fatality rate decreased from 6% to 3% (Figure 4).

**Figure 5.**
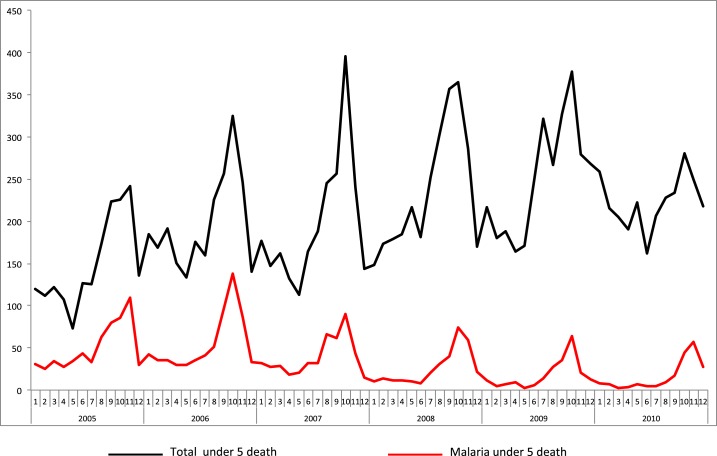
Reported deaths due to confirmed malaria and to all causes in public health facilities in Senegal, 2005–2010. Source: NMCP, Senegal.

Although the total number of deaths reported by health facilities did not change between 2005 and 2010, from 2007 on, the number of deaths due to malaria decreased while still maintaining seasonal peaks that coincided with expected trends in malaria transmission (Figure 5). These data represent patients hospitalized at district health centers (*N* = 76) and publicly supported referral hospitals (*N* = 23), with near total completeness of reporting.

### Change in contextual factors.

#### Economic growth.

Annual gross domestic product per capita in Senegal increased from USD$492 to USD$1,055 over the past decade.

#### Other health interventions.

During the period 2005–2010, the proportion of children who received ORS for treatment of diarrhea increased from 15% to 22%. Complete immunization coverage increased from 58% in 2005 to 64% in 2010 (*P* < 0.001). Early initiation of breastfeeding increased from 23% to 48% (*P* < 0.001). Vitamin A supplementation increased from 74% to 79% (*P* < 0.001). The prevalence of pneumonia decreased from 13% to 5%, whereas the prevalence of diarrhea remained similar, at 22% and 21%, respectively, in 2005 and 2010. However, nutritional indicators deteriorated, with an increase in stunting from 16% to 26%, and an increase in wasting from 7% to 10% (Table 7).

**Table 7:** Evolution of contextual factors related to child health in Senegal 2005–2010

Factors	DHS 2005		DHS 2010		Absolute change % (95% CI)	*P* value
	% (95% CI)	*N*	% (95% CI)	*N*		
IMCI coverage						
Oral rehydration salts for diarrhea (ORS)	15.1 (13.4–16.6)	2,168	22 (20.3–23.7)	2,243	6.9 (4.6–9.2)	0.001
Vaccine coverage						
BCG	91.7 (90.5–92.9)	2,024	94.7 (93.7–95.6)	2,199	3.0 (1.4–4.5)	0.001
DPT3	78.3 (75.5–80.1)	2,024	82.6 (81.0–84.2)	2,199	4.3 (1.9–6.7)	0.001
Polio3	72.9 (70.9–74.8)	2,024	72.7 (70.8–74.5)	2,199	−0.2 (–2.8;2.5)	0.442
Measles	73.9 (71.9–75.8)	2,024	82.1 (80.5–83.7)	2,199	8.2 (5.7–10.7)	0.001
Complete vaccine coverage	58.0 (55.9–60.0)	2,024	63.8 (61.7–65.9)	2,199	5.8 (-8.7; −2.8)	0.001
Micronutrient supplementation						
Vitamin A supplementation	74.4 (73.5–75.2)	10,077	79.3 (78.5–73.5)	11,633	4.9 (3.7–6.0)	0.001
Nutritional status						
Stunting	16.3 (14.9–17.6)	2,883	26.5 (25.1–27.9)	3,761	10.2 (8.2–12.1)	0.001
Underweight	17.3 (15.9–18.7)	2,883	17.7 (16.5–18.9)	3,761	0.4 (−1.4; 2.2)	0.33
Wasting	7.6 (6.7–8.4)	2,883	10.1 (9.0–11.2)	3,761	−2.5 (–3.8; −1.1)	0.001
Breastfeeding						
Early initiation of breastfeeding	22.7 (21.6–23.7)	6,221	48.0 (46.5–49.4)	4,509	25.3 (23.5–27.1)	0.001
Exclusive breastfeeding for < 6 months	34.1 (32.9–35.2)	6,221	39.0 (37.5–40.4)	4,509	4.9 (3.0–6.7)	0.001
Other Childhood Illnesses						
Prevalence of diarrhea	22.3 (21.4–23.1)	9,709	20.6 (19.4–21.3)	10,893	−1.7 (−2.8; −0.5)	0.001
Prevalence of ARI	13.2 (13.5–13.8)	9,709	5.4 (4.9–5.8)	10,893	−7.8 (−8.5; −7.0)	0.001

Source: DHS 2005, 2010. ARI = acute respiratory infections.

#### Environmental influences on malaria.

**Figure 6.**
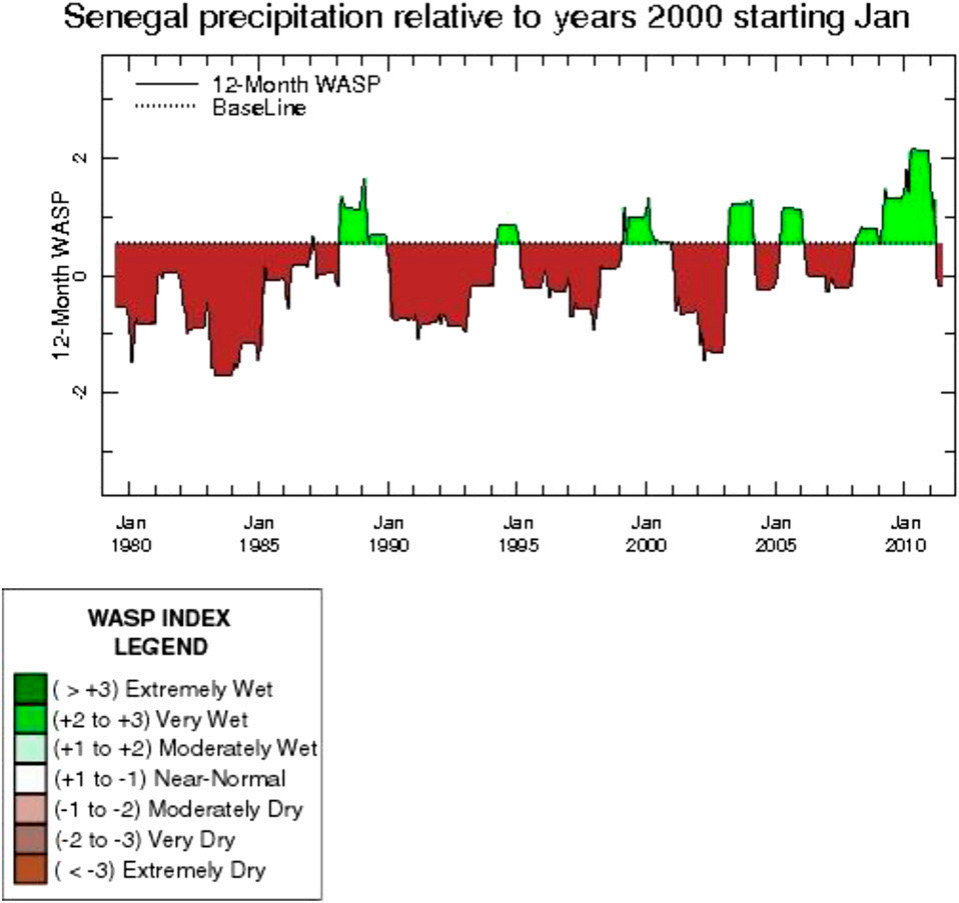
Weighted Anomaly Standardized Precipitation (WASP) Index for Senegal 1980–2010. Source: IRI, 2014.

Environmental influences were measured in two ways; the NDVI†, and the WASP index‡.^15^ Historically, from 1950 until 2005, annual rainfall in Senegal has decreased by about 30% and temperatures have increased according to the Center de Suivi Ecologique. However, since 2005, average annual rainfall increased substantially. From 2000 to 2005, Senegal experienced relatively dry conditions in the majority of the country. The WASP index shows that rainfall during the 2000–2005 period was moderately dry as compared with 2000. During 2006–2007, there was substantial regional variability, though for most regions 2007 was a relatively dry year. From 2008–2010, rainfall was substantially higher than average throughout the country, as indicated by a WASP index ranging from 0.5 to 2.0 over the baseline (Figure 6). In the zone of moderate malaria parasite prevalence (central Senegal: Fatick, Kaffrine, Kaolack, and Ziguinchor), positive rainfall anomalies were observed. In Kaolack, positive rainfall anomalies were observed, averaging 129 mm over baseline between 2005 and 2010. In Ziguinchor, during the same time frame, positive rainfall anomalies of 200 mm and more were recorded. In the area of high malaria prevalence (southeastern Senegal: Sédhiou, Kolda, Tambacounda, and Kédougou), positive rainfall anomalies were also confirmed. In Tambacounda, positive anomalies of 80–300 mm were recorded between 2003 and 2010. In Kolda, positive anomalies of 50–500 mm were recorded in the same timeframe. These positive rainfall anomalies correspond to an increase in vegetation, particularly during 2010. Overall, rainfall, temperature, and vegetation indices from 2006 to 2010 favored a maintenance or increase of malaria transmission.

## DISCUSSION

After considering the potential effects of many contextual factors such as environmental trends or other maternal and child health interventions, this evaluation has determined that it is plausible that effective malaria control contributed significantly to the impressive gains in child survival demonstrated by Senegal between 2005 and 2010.

The plausibility argument set forth by Rowe and others^3^ and adopted by the RBM Monitoring and Evaluation Reference Group suggests the impact of malaria control can be inferred when data show a scale-up of interventions and a subsequent decline in ACCM, while considering or controlling for contextual factors. This plausibility can be further substantiated when the relationship between interventions and mortality decline holds up across different populations and epidemiologic zones.

At the national level in Senegal, coverage of the major malaria control interventions was expanded considerably between 2005 and 2010. Household ownership of ITNs increased from 20% of households to 63%, and the proportion of the population sleeping under an ITN likewise increased from 6% of the population to 29%. Coverage of both IRS and IPTp treatment during pregnancy, while protecting smaller populations, both increased substantially from 2005 to 2010. Although improvement in case management is difficult to measure using household surveys in a low incidence country during scale-up of routine use of parasitologic diagnostic tools, health facility data collected by the NMCP indicated that more than 90% of suspected cases received a diagnostic test in 2010.

In this same time frame, at the national level, Senegal saw a 50% decline in parasite prevalence and a 40% decline in ACCM, while surveillance data showed a decrease in proportion of consultations due to malaria from 34% to 3%, and a decline in proportion of deaths due to malaria from 19% to 4%. To some degree, the decline in malaria-specific cases and deaths may be related to introduction and rapid uptake of RDTs and change in case definition to require parasitologic confirmation. However, the consistent declines across these impact indicators suggest Senegal was successful in controlling malaria over the 5 year period.

Considering contextual factors supports the hypothesis that malaria interventions contributed to a decrease in ACCM as it is not clear these other factors could account for all of the decrease in mortality. Although economic growth in the country may have contributed to the decline in child mortality, the causal pathway is less well-defined and this study did not attempt to measure this relationship. Improvement in economic and sociodemographic indicators in the communities may have contributed to progress in maternal and child health, as illustrated by progress in ORS utilization and immunization rates among children. Although there was a substantial reduction in the proportion of children under five who had symptoms of pneumonia in the previous 2 weeks, possibly due in part to the introduction of *Haemophilus influenza* b vaccine in 2005, the proportion with diarrhea did not change, and the gains may have been offset by worsening nutritional status. Although most of the changes in the contextual factors were positive, with the exception of nutritional status, the magnitude of positive change in intervention coverage levels for other child survival activities is relatively small, and does not match the magnitude of the increase of malaria control activities. The increase of key interventions for malaria control appears as the most likely factor to explain the observed reductions.

The relationship between intervention coverage and morbidity and mortality declines is further supported when the data were analyzed by epidemiologic zones. Central and southern Senegal are the zones with the highest transmission of malaria, where one would expect to see higher malaria-related morbidity and mortality. Following expanded coverage with ITNs, the surveys show higher ownership and use of ITNs in these same zones, particularly among children under five (Figure 3B). Following the mass distributions of ITNs, the levels of parasitemia in children under five fell from 7% to 4% in the center zone and 21% to 8% in the south, and ACCM fell from 123/1,00 live births to 68/1,000 live births in the center and from 165/1,000 live births to 96/1,000 live births in the south, zones with the greatest potential to benefit from increased intervention coverage. Routine health facility data on malaria also demonstrated substantial decreases in the number of confirmed malaria cases among children under five in the southern zone, while remaining stable in the rest of the country.

The data were also analyzed by wealth quintile to look at the impact of malaria control on the poorest populations. Although there were substantial changes in ITN ownership across all wealth quintiles in the analysis, the largest increases were seen in the two poorest categories, where greater than 75% of homes owned at least one ITN by 2010. High levels of ownership translated into high levels of use in these same two lowest wealth quintiles. Among children under five, there was a significantly greater increase in ITN use among the poorest populations than that seen among the wealthy (Figure 3A). The impact of this dramatic increase of ITN use among the poor resulted in a corresponding decline in parasitemia in the same subpopulations. Parasitemia in children under five declined from 16% to 6% among the poorest children, and from 7% to 2% among the next poorest group, as compared with the middle and wealthiest quintiles where declines were not observed. Declines in ACCM were also greatest among the three poorest quintiles, with percentage reductions in ACCM of greater than 40% in all three groups. The proportionally greater reductions in both morbidity and mortality among the socioeconomic groups who also benefited from the greatest increases in ownership and use of ITNs contribute to the evidence that malaria control influenced impact on child health in Senegal.

Despite the large increases in intervention coverage from 2005 to 2010, and distribution of ITNs in such a way that increased access most for the poorest and those at highest risk, intervention coverage remained suboptimal in 2010. Subsequent cross-sectional surveys have demonstrated slow but steady improvements in ITN access, IPTp, and diagnosis with RDTs since 2010.

Limitations of this study include those inherent to this evaluation design, including the timing of measuring intervention coverage to mortality measures, no quantification of malaria control’s contribution to declines in all-cause mortality, and factors potentially affecting ACCM which were not covered in our data sources. There are some limitations which may be specific to Senegal. The case definition changed from clinical to parasitologically confirmed during the period of the evaluation, with a suspect case defined as one without clinical evidence for an alternate fever source (resulting in a lower proportion of children with fever to be tested according to policy). In addition, in a low burden country and in the context of rapidly increasing use of diagnostic confirmation, very few febrile children who are tested test positive, and thus the indicator of proportion of children under five with fever in the past 2 weeks who received an antimalarial should be low and show a decreasing trend if providers are adhering to test results, making it difficult to ascertain the contribution of correct case management.

During the period 2005–2010, Senegal successfully expanded coverage of four proven interventions for malaria control, simultaneously achieving higher levels of coverage of preventive interventions, and improving access to prompt and appropriate care and treatment. During that same period, ACCM declined significantly on a national level, and declines were greater among the epidemiologic zones and socioeconomic quintiles that are most at risk for malaria and have the greatest potential to benefit from improved access to these interventions. After considering the potential effects of many contextual factors such as environmental trends or other maternal and child health interventions, it is plausible that effective malaria control contributed significantly to the impressive gains in child survival demonstrated by Senegal between 2005 and 2010.

## Footnotes

† The Normalized Difference Vegetation Index (NDVI) is the ratio between the difference of red and near-Infrared (NIR) divided by the sum of red and near-infrared reflectances. The index provides some information on healthy vegetation by examining their difference in wavelength absorption and reflectance. Healthy vegetation growth, such as forests, will yield high NDVI values closer to 1 while low vegetation will yield values close to 0.2.

‡ To compute the WASP index, monthly precipitation departures from the long-term (30-year) average are obtained and then standardized by dividing by the standard deviation of monthly precipitation. The standardized monthly anomalies are then weighted by multiplying by the fraction of the average annual precipitation for the given month. These weighted anomalies are then summed over a 12-month period.
